# Graphene Oxide Incorporated Forward Osmosis Membranes With Enhanced Desalination Performance and Chlorine Resistance

**DOI:** 10.3389/fchem.2019.00877

**Published:** 2020-01-10

**Authors:** Zhanguo Li, Yi Wang, Mengwei Han, Dayong Wang, Shitong Han, Zequn Liu, Ningyu Zhou, Ran Shang, Chaoxin Xie

**Affiliations:** ^1^State Key Lab of NBC Protect for Civilian, Beijing, China; ^2^Water Industry and Environment Engineering Technology Research Centre, Chongqing, China; ^3^Service Bureau of Agency for Offices Administration of the CMC, Beijing, China; ^4^Department of Military Facilities, Army Logistics University, Chongqing, China

**Keywords:** forward osmosis, GO nano-sheets, thin-film composite membrane, desalination performance, chlorine resistance

## Abstract

In this work, grapheme oxide (GO) nano-sheets were synthesized and dispersed in the aqueous phase for the interfacial polymerization (IP) process to develop a new type of thin-film composite (TFC) membranes for forward osmosis (FO) applications. The effects of the GO concentrations on the membrane surfaces and cross-sectional morphologies and FO desalination performances of the as-prepared TFC membranes were investigated systematically. Compared with the control membrane, the optimal GO-incorporated TFC membrane displayed higher water flux, less specific reverse solute flux (SRSF) and lower structure parameter. Moreover, the optimized membrane showed 75.0 times higher chlorine resistance than the control membrane. In general, these new type of membranes could be an effective strategy to fabricate high-performance FO membranes with good desalination performance and chlorine resistance.

## Introduction

Desalination refers to the process of removing salts or minerals dissolved in seawater or brackish water to obtain water for human and animal consumption, irrigation and industrial process, which is a significant technology can effectively alleviate the lack of fresh water for the whole global (Marcovecchio et al., 2005). Common desalination methods include thermal methods represented by multi-stage flashing and multi-effect evaporation and membrane-based techniques represented by electro-dialysis, nano-filtration (NF) and reverse osmosis (RO) (Darwish and ElDessouky, 1996; Khawaji et al., 2008). Among them, RO technology is currently the most popular technology and more than 50% of the world's 15,000 desalination plants are RO-technology-based (Greenlee et al., 2009).

Despite the wide application of RO, this technology has some disadvantages such high energy consumption, low water recovery and severe brine pollution. Forward osmosis (FO) technology has been widely concerned by researchers and industrial circles recently. This technology utilizes the difference of osmotic pressure (or chemical potential) between a low-concentration feed solution (FS) and a high-concentration draw solution (DS) to drive water molecules across a semi-permeable membranes (Zhao et al., 2012). Compared with RO, the FO technology has shown advantages, such as low energy input of operation (Mazlan et al., 2016), efficient rejection of contaminants (She et al., 2013; Kong et al., 2014), and less propensity of membrane fouling (Emadzadeh et al., 2014a; Salehi et al., 2017). More recently, polyamide thin film composite (PA-TFC) membranes have been widely studied due to their high mechanical strength, satisfactory salt rejection and hydrolytic stability (Sukitpaneenit and Chung, 2012; Han et al., 2016).

Although PA-TFC membranes possess many advantages, its resistance to disinfectants with chlorine needs to be further improved for FO's wide practical applications. This is because the pervasive biological fouling is the major cause decreases membrane performance (Mi and Elimelech, 2010; Xue et al., 2016). Thus, disinfectants with chlorine for controlling bio-fouling or cleaning agents are needed in water treatment process, which can result in changes of the polyamide chains by the N-chlorination, Orton rearrangement, and directing chlorination reactions (Verbeke et al., 2017), consequently cause the sharp decline in membrane salt rejection. Apart from this, conventional PA-TFC membranes also face the problem of low water fluxes ascribed to their relatively hydrophobic and thick surfaces (Emadzadeh et al., 2013; Ma et al., 2017). The water molecules need to pass through the PA layer, thus its thick thickness and hydrophobicity make water transfer lower due to the increased membrane mass transfer resistance. It has been proved that the thinner and more hydrophilic PA layer improves the membrane water permeability of the PA-TFC membrane (Han et al., 2012; Wang et al., 2016). Based on the above two issues, PA-TFC membranes with improved chlorine resistance and thin active layer are highly demanded.

Many efforts have been devoted to improve these performances of FO PA-TFC membranes. Recently, various nano-materials have been widely studied to be incorporated into the PA layer to enhance the TFC membranes performances and showed great performance improvements, such as nano-silver, nano-titanium dioxide, nano-silica and hydroxide nanoparticle, etc. (Emadzadeh et al., 2014b; Niksefat et al., 2014; Liu and Hu, 2016; Lu et al., 2016, 2018). Among them, grapheme oxide (GO), a promising two-dimensional carbon nano-material, has attracted great attention in the material research field. Its excellent physical properties coupled with flexibility in chemical functionalization ascribed to the abundant oxygen-containing functional groups, make GO an excellent candidate for various applications (Hu and Mi, 2013). Mariana Ionita et al. added GO in the preparation of ultrafiltration polysulfone (PSf) membrane to increase the water flux of the membrane. At the same time, the thermal stabilities and mechanical properties of these GO-incorporated PSf membranes were also significantly improved, but the required GO concentration was as high as 1.0 wt% (Ionita et al., 2015). Ho Kyong Shon's group studied similar PSf-GO based substrate for interfacial polymerization to form the PA layer of TFC FO membranes. Results revealed that at a relatively low amount of GO addition (0.25 wt%), the as-prepared membranes showed not only significantly improved water permeability but also effective PA layer formation (Park et al., 2015). In addition, Hee-Ro Chae et al. added only 76 ppm GO to the aqueous phase of the interfacial polymerization (IP) process to prepare RO membranes, these membranes possessed improved water flux, salt rejection as well as anti-fouling properties (Ali et al., 2016).

Inspired by these outstanding work, in this paper, we first applied low-concentration GO nano-sheets in IP process for FO membranes fabrication. Mainly based on the following three reasons: Firstly, due to the two-dimensional capillary effect of GO nano-sheets, the water flux of the GO-incorporated FO membrane can be improved while remaining the salt rejection performance (Ali et al., 2016); Secondly, the abundant hydrophilic functional groups in the GO nano-sheets would increase the membrane water flux and thus desalination performance; Finally, the PA active layer would be protected by GO nano-sheets thus improved its chlorine resistance. The FO membranes were synthesized by adding different concentrations of GO nano-sheets into the aqueous phase of IP process. A series of characterizations were conducted to understand the influence of GO nano-sheets on the active layer morphology and hydrophilicity. FO membrane experiments were performed to evaluate the desalination performance and chlorine resistance as well as determine the most suitable GO nano-sheets incorporation concentration.

## Experimental

### Materials

PSf with 22,000 Da average molecular weight (Sigma-Aldrich, USA), polyvinylpyrrolidone (PVP) (K 30, Sigma-Aldrich, USA), 1-methyl-2-pyrrolidinone (NMP) (99.5%, Sigma-Aldrich, USA) and N-N-Dimethylformamide (DMF) (99.8%, Sigma-Aldrich, USA) were used for PSf ultrafiltration substrates preparation. m-Phenylenediamine (MPD) flakes (99%, Aldrich, China) and 1,3,5-Benzenetricarbonyl chloride (TMC) (98%, Sigma-Aldrich, USA) dispersed in hexane (98%, Aldrich, China) were used for the IP process. Graphite (Sigma-Aldrich, USA), sodium nitrate (NaNO_3_) (Beijing Chemical Factory, China), hydrogen peroxide (H_2_O_2_) (Beijing Chemical Factory, China), sulfuric acid (H_2_SO_4_) (Beijing Chemical Factory, China), and potassium permanganate (KMnO_4_) (Sigma-Aldrich, USA) were used for GO nano-sheets preparation. For membranes chlorine resistance tests, sodium hypochlorite (NaClO) (Chemical Supply Pty Ltd, China) was used. Sodium chloride (NaCl) (ACS reagent, Beijing Chemical Factory, China) was dissolved in deionized water (DI) produced with a Milli-Q system (Millipore, USA) for FO tests.

### Methods

Graphene oxide (GO) was synthesized from graphite powders by the classical Hummer's method (Hummers and Offeman, 1958; Hirata et al., 2004; Park and Ruoff, 2009). 1.0 g graphite and 0.5 g NaNO_3_ were added into an Erlenmeyer flask with 23.0 ml concentrated H_2_SO_4_ solution, and then stirred under an ice bath. After that, 3.0 g KMnO_4_ was added slowly for 2.0 h, then transferred the mixture into a 35.0°C water bath and continued to stir for 0.5 h. Then 46.0 ml DI water was slowly added, and the reaction was continued for 0.5 h as the temperature raised to 98.0°C. Finally, 140.0 mL DI water and 10.0 mL of H_2_O_2_ solution were added to the obtained solution to terminate the reaction. The mixture was filtered and then washed thrice with DI water to obtain the final product. Then, the as-prepared GO nano-sheets were washed three times with DI and methanol, respectively, to remove the unreacted chemical residuals. Finally, the obtained powders were freeze-dried for 48.0 h to obtain the GO nano-sheets.

Crystal structure of the synthesized GO nano-sheets was tested via a Miniflex 600 diffractometer (Rigaku, Japan) powder X-ray diffractometer (PXRD) in the 2θ range of 2–50° at room temperature. Morphologies of the GO nano-sheets were taken with a field-emission scanning electron microscope (Merlin ZEISS GEMINI2) operating at 5 kV and 13 pA. Attenuated total reflection Fourier transform infrared (ATR-FTIR, Thermo Scientific Nicolet 6700) was used to characterize the GO nano-sheets. The XPS (VG Scientific, UK) measurements were also carried out for GO nano-sheets characterization using Mg Kα radiation (hυ = 1,253.6 eV). The C 1s spectra were recorded with the pass energy Ep = 20 eV whereas the wide scans with Ep = 100 eV.

### The Preparation of Polysufone (PSf) Substrate

Flat-sheet PSf substrates were prepared by using a mixed PSf-PVP casting dope. 16.5 g PSf, 0.5 g PVP, 21.0 g NMP and 62.0 g DMF were magnetically stirred for at least 24.0 h and then left degassing for 8.0 h to prepare the casting dope. A thin layer (100 ± 10 μm) of the obtained dope was cast on a clean glass plate via a casting machine (Wu Han Zuoneng Instruments Ltd., China). The whole composite was exposed in the air for 10.0 s followed by immersing into a DI water bath to initiate phase inversion process. Once the substrate was peeled off from the glass plate, it was removed from the water bath, thoroughly rinsed with DI water for three times and then transferred to a 4°C DI water bath for storage till later use (Qin et al., 2013; Shen et al., 2015; Yu et al., 2019).

### The Preparation and Characterization of Control and GO-Incorporated TFC Membrane

**Figure 1:**
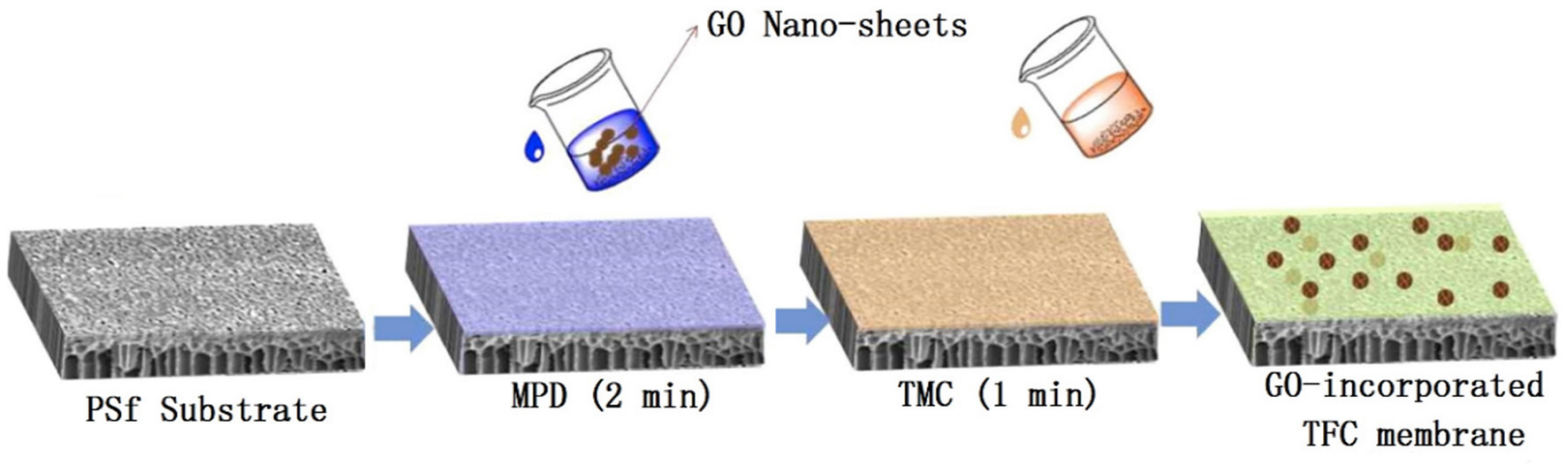
The preparation procedures of GO-incorporated TFC membranes.

The synthesis procedure of the TFC-control membrane without GO was below (Wang et al., 2018a). The PSf substrate was first immersed in an aqueous solution containing 3.4 wt% MPD monomers for 2.0 min. Excess MPD solution was removed by a rubber roller. After that, the substrate was immersed into a 0.15 wt% TMC solution for 1.0 min to form the PA layer. The fabrication of GO-incorporated membranes was similar to that of TFC-control membrane, except that GO nano-sheets were added in the aqueous solution before IP process, as shown in Figure 1 and Table 1. GO with various concentrations from 0.0025 to 0.02 wt% was dissolved in aqueous solution by ultra-sonication under an ice bath for 30.0 min. The GO/MPD-saturated substrates were immediately placed to react with the TMC/n-hexane solution for the formation PA layers. The synthesized GO-incorporated membranes were denoted by GO-1, GO-2, GO-3, GO-4, and GO-5, corresponding to a GO loading of 0.0025, 0.0050, 0.0100, 0.0150, and 0.0200 wt%, respectively. The as-prepared membranes were also rinsed with DI water thoroughly for three times and then stored in DI water at 4°C for later tests.

**Table 1:** Reagents for TFC series membranes preparation.

**Membranes**	**MPD (wt%) in aqueous phase**	**GO (wt%) in aqueous phase**	**MPD/GO solutions viscosity (cp)**	**TMC in organic phase (wt%)**
TFC-control	3.4	0	60.00	0.15
GO-1	3.4	0.0025	60.67	0.15
GO-2	3.4	0.0050	60.67	0.15
GO-3	3.4	0.0100	61.33	0.15
GO-4	3.4	0.0150	62.00	0.15
GO-5	3.4	0.0200	62.67	0.15

The morphologies of the TFC membranes surfaces and cross-sections (fractured in liquid nitrogen) were observed by FESEM (Merlin ZEISS GEMINI2). Fifteen random tests were conducted based on three different batch membranes in cross-sectional images for PA layer thickness tests. Water contact angles were measured using a contact angle system (Dataphysics OCA 20). For each membrane, ten measurements were performed for each of three independently prepared membranes. ATR-FTIR spectroscopy instrument (Thermo Scientific Nicolet 6700) was used to confirm the change of the PA layer functional groups.

### Evaluation of TFC Membrane Desalination Performance

All the as-prepared membranes were tested in a lab-scale test system, which has been described in our previous work (Wang et al., 2018a,b). The co-current cross-flow velocity was 4.9 cm/s for both the feed and draw solutions. The temperatures of the feed and draw solutions were maintained at 20 ± 0.5°C. 1.0 and 2.0 M NaCl solutions were used as the DSs and a balance (ML4002, METTLER TOLEDO) was connected to a computer to record weight changes of the permeated water at 30.0 s intervals. 10.0 mmol L^−1^ NaCl solution were used as the feed solution (FS) and a conductivity meter was used to calculate the reverse solute flux of the membrane at 60.0 s intervals. Membranes were tested under two different modes: (1) active layer facing draw solution (AL-DS) mode; and (2) active layer facing feed solution (AL-FS) mode. Every test was conducted for 1.0 h in triplicate. The water permeation flux (Jw) (L • m^−2^h^−1^, LMH), reverse solute flux (Js) (g • m^−2^h^−1^), and specific reverse solute flux (SRSF) (g • L^−1^) were calculated by the same methods detailed in our previous work (Wang et al., 2018a,b).

### Evaluation of Membrane and Chlorine Resistance

The chlorine resistance of the tested membranes was evaluated by exposing their top surfaces to a 1,000 ppm NaClO solution over different periods. The NaClO solutions were kept in dark and replaced every 2.0 h during the test to maintain a constant concentration. To access the as-prepared membranes chloride resistance quantitatively, a doubled increase value of SRSF was selected as an upper limit, indicating the PA layers were degraded dramatically by the active chlorine to be unacceptable in FO processes (Lu et al., 2017; Wang et al., 2018c). Before the membrane FO performance tests, these membranes were removed from NaClO solutions and then washed 3 times with DI water to avoid the residual chlorine oxidation during tests.

### Determination of FO Membrane Transport and Structural Parameters

The transport and structural parameters of these as-prepared membranes, the water permeability coefficient (A), salt permeability coefficient (B), and structural parameter (S) were tested by a method developed by Elimelech's Group (Tiraferri et al., 2013). The method includes a four-stage test, every stage using a DS with different concentration. The water permeation flux (Jw) and reverse solute flux (Js) were measured through non-linear regression based on Equations (1) and (2), in every FO test stage. The presented data were the average values based on triplicated measurements.

(1) $$\begin{array}{l} \text{Jw }=\text{ A}\left\{\frac{\pi_{D}\;exp\left(-\frac{J_{w}S}{D}\right)-\pi_{F}}{1+\frac{B}{J_{w}}\left[1-exp\left(-\frac{J_{w}S}{D}\right)\right]}\right\} \end{array}$$

(2) $$\begin{array}{l} \text{Js }=\text{ B }\left\{\frac{C_{D}\;exp\left(-\frac{J_{w}S}{D}\right)-C_{F}}{1+\frac{B}{J_{w}}\left[1-exp\left(-\frac{J_{w}S}{D}\right)\right]}\right\} \end{array}$$

where D is the bulk diffusion coefficient of the draw solute.

## Results and Discussions

### GO Nano-Sheets Characteristics

**Figure 2:**
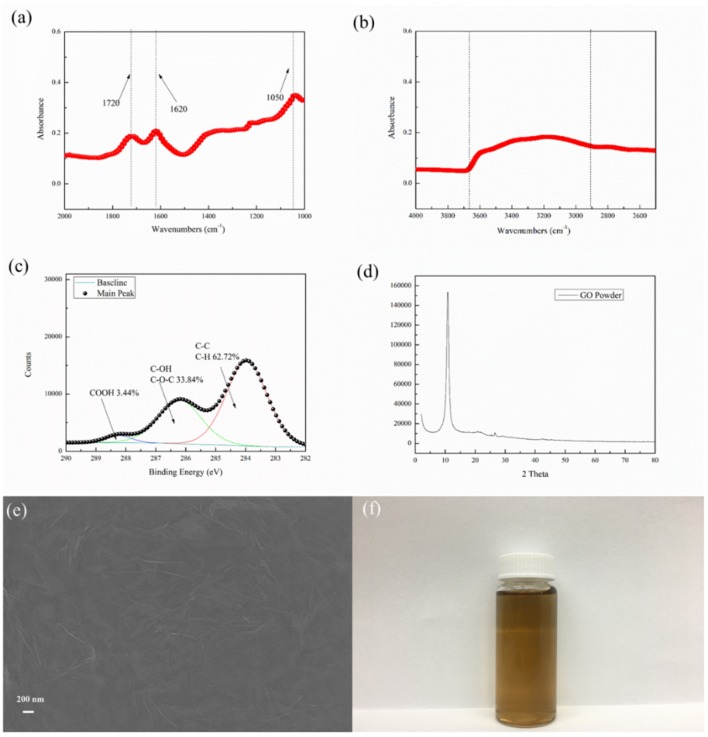
GO nano-sheets characteristics **(a), (b)** ATR-FTIR spectra, **(c)** High-resolution XPS spectra of C 1s, **(d)** XRD, **(e)** SEM images and **(f)** GO nano-sheets water solution.

The ATR-FTIR spectra for GO nano-sheets were presented Figures 2a,b. The presence of C–O bonds (1,050 cm^−1^) in epoxy groups, C=C bonds (1,620 cm^−1^) in unoxidized carbon crystal lattice, and C=O bonds (1,720 cm^−1^) in carboxyl groups, can be observed in the synthesized GO nano-sheets, similar to Jiang's work (Jiang et al., 2014). Meanwhile, an intense band between 3,100 and 3,600 cm^−1^ indicated that GO nano-sheets with many free hydroxyl groups can bridge hydrogen bonds and enhance intermolecular forces between GO nano-sheets and PA polymer chains. Moreover, the analysis of the chemical valence of elements can be used to estimate the oxidation degree of GO nano-sheets (Stankovich et al., 2007; Stoller et al., 2008; Zhou et al., 2010). The high-resolution XPS C 1s spectra of the GO were shown in Figure 2c, the peaks of 284.0, 286.2, and 288.3 eV can be attributed to C–C, C–H, C–O, and C=O groups, respectively. According to these peak areas, it is estimated that about 60% of C didn't undergo oxidation reaction, 33.84% of oxidized C contained C–O and 3.44% of oxidized C contained COOH. Moreover, the XRD pattern and SEM image of the GO nano-sheets were shown in Figures 2d,e, which are consistent with Leszek Stobinski et al.'s results (Stobinski et al., 2014). Finally, after the graphene oxidation, new oxygen-containing groups were formed on the surface, such as C=O, C–OH, C–O–C, etc., thus the aqueous GO solution (see Figure 2f) showed a typical light yellow color, which is similar to the reported characteristics in the literature (Masuda et al., 2002; Novoselov et al., 2004; Allen et al., 2009). Based on, the GO nano-sheets were successfully synthesized.

### Membrane Characterization

#### Membrane Morphology

**Figure 3:**
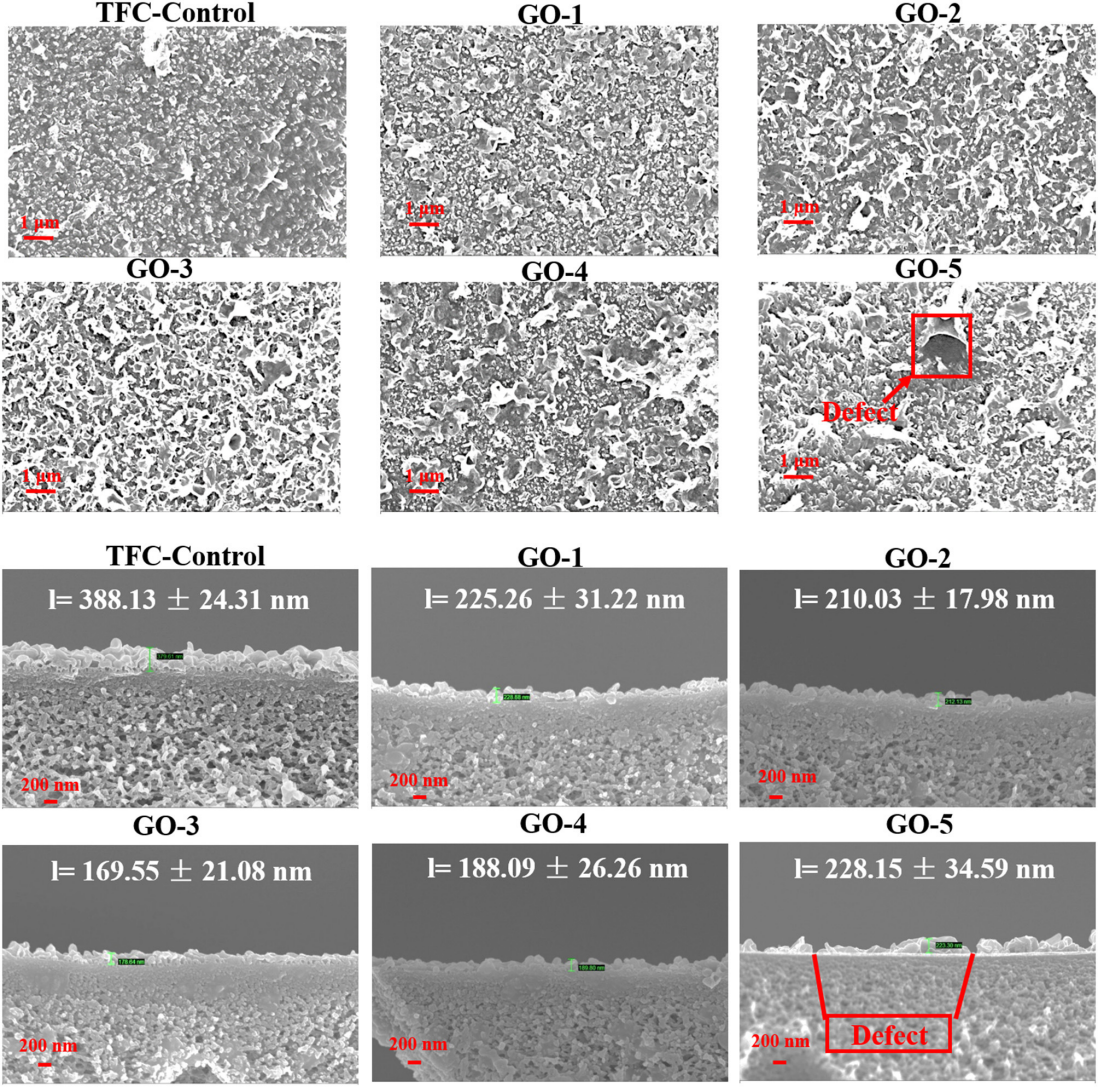
SEM images of TFC-control and GO-incorporated series membranes, in which l means PA layer thickness.

The surface and cross-section morphologies of the TFC-control and GO-incorporated series TFC membranes were investigated, as shown in Figure 3. All the membranes surfaces possessed “ridge and valley” or “flower” characteristics, which is the typical morphology of the PA membrane. Additional thin layers were observed on top PSf substrates via the cross-sectional SEM images, which confirms the successful reaction of interfacial polymerizations. Obviously, the incorporation of GO nano-sheets into the active PA layer can significantly affect the membrane surface as well as cross-sectional morphology. Compared to the pristine membrane, all the GO-incorporated membranes displayed denser and relatively smoother surfaces. Meanwhile, the PA layer thickness of the control TFC membrane was much higher than those of GO-incorporated TFC membranes. For example, GO-3 membrane had PA layer thickness of 169.55 nm, which is only about 1/2 of the control-TFC membrane. However, when the concentration of GO in the aqueous phase increased to 0.0200 wt%, defects would be observed on the membrane surface as well as membrane cross section. At the same time, the PA layer thickness increased slightly compared to the control membrane, which might compromise membrane FO selectivity.

**Figure 4:**
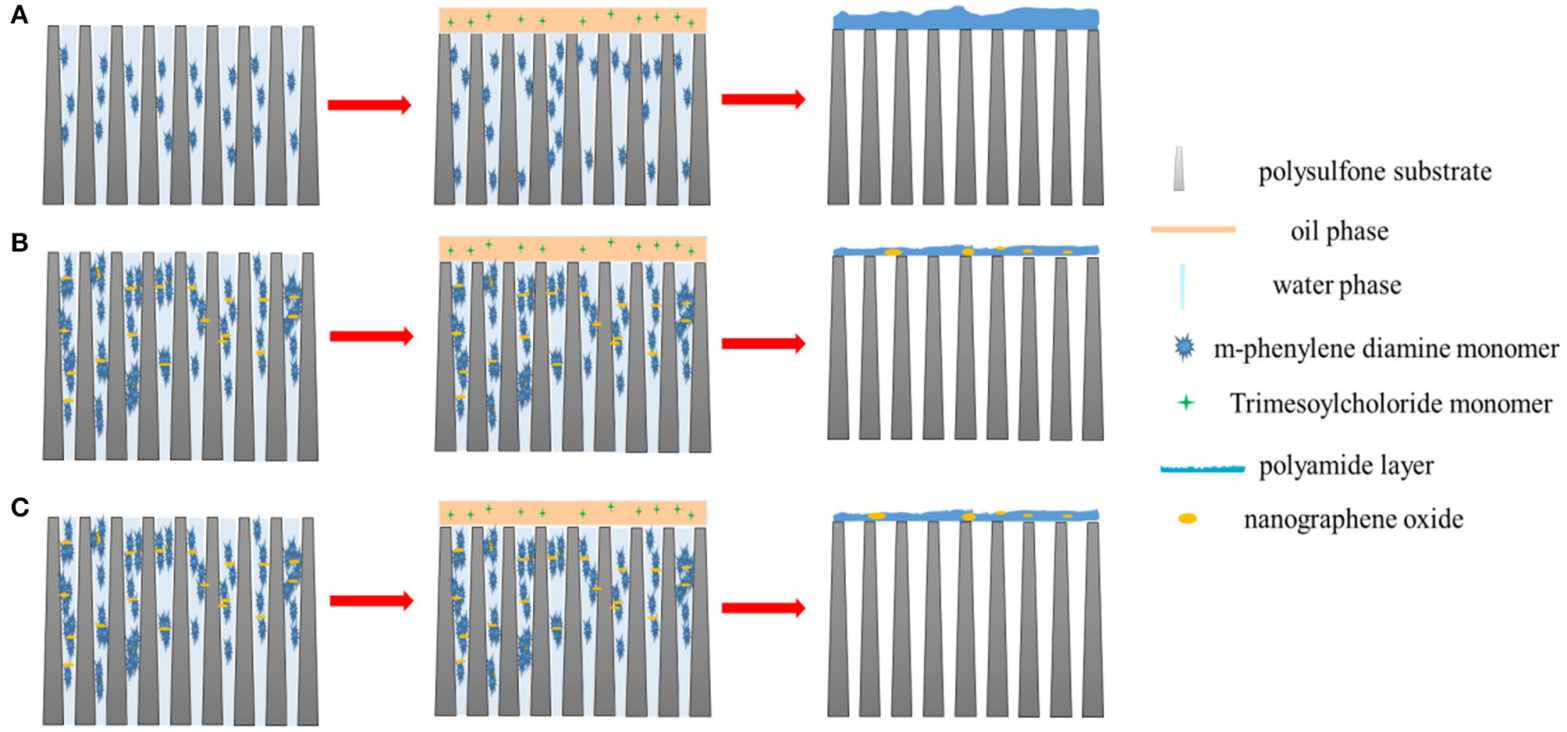
Schematic diagram of **(A)** the conventional interfacial polymerization protocol, **(B)** the low-concentration GO nano-sheets influenced interfacial polymerization protocol and **(C)** the high-concentration GO nano-sheets influenced interfacial polymerization protocol.

The remarkable changes of PA layers in GO-incorporated membranes may be contributed by the following reasons, as shown in Figure 4. Generally, TMC monomers are less soluble in the water phase, thus IP reaction happens in the organic phase. MPD monomers need to diffuse from the water phase to the organic phase to react with the TMC monomers (Luo et al., 2014). The substrate was saturated by the MPD/GO aqueous solution vertically, GO nano-sheets tend to position along the membrane surface horizontally due to the Langmuir–Blodgett film deposition (Chae et al., 2015). The horizontal incorporation of GO nano-sheets would interfere the diffusion of MPD monomers, resulted in smoother active PA layers. In the meanwhile, hydrogen bonds presented in the hydroxyl groups of GO can contribute to a more compact chain structure. Moreover, these nano-sheets incorporation decreased the amount of MPD monomers and increased the viscosity of the aqueous solution (see Table 1), thus limited the reaction between MPD and TMC monomers and formed thinner active layers. However, when the loading in the aqueous phase increased to 0.0200 wt%, which caused the serve agglomerations of the GO nano-sheets, thus formed a defective interface enables MPD to further diffuse through the pristine PA layer, resulting in a slightly thicker PA layer.

#### Membrane Hydropholicity

**Figure 5:**
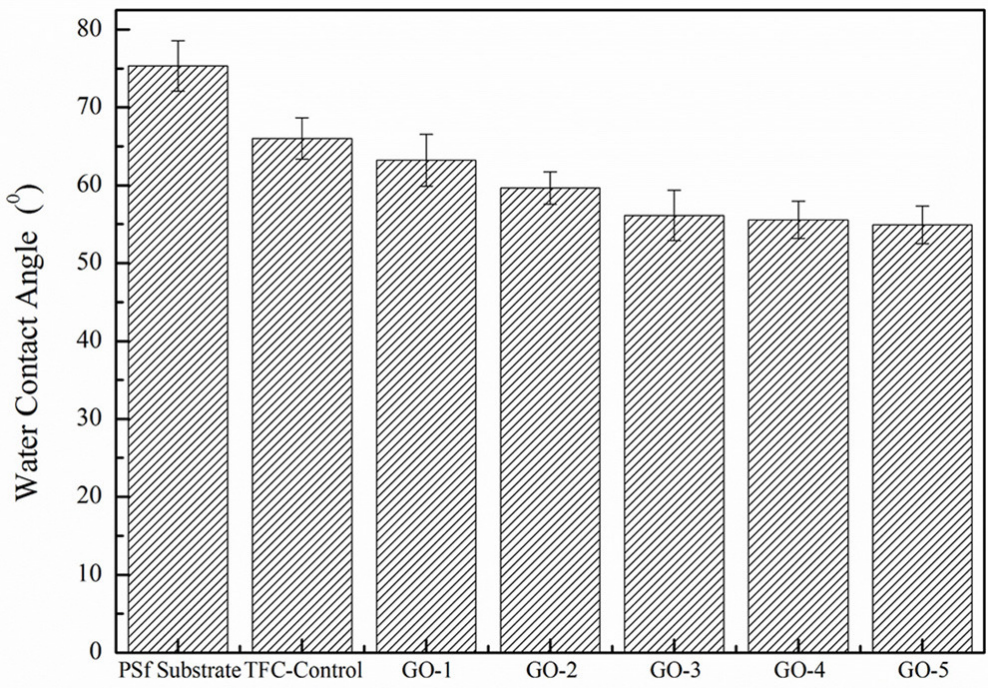
Water contact angles of PSf substrate, TFC-Control and GO-incorporated membranes.

**Figure 6:**
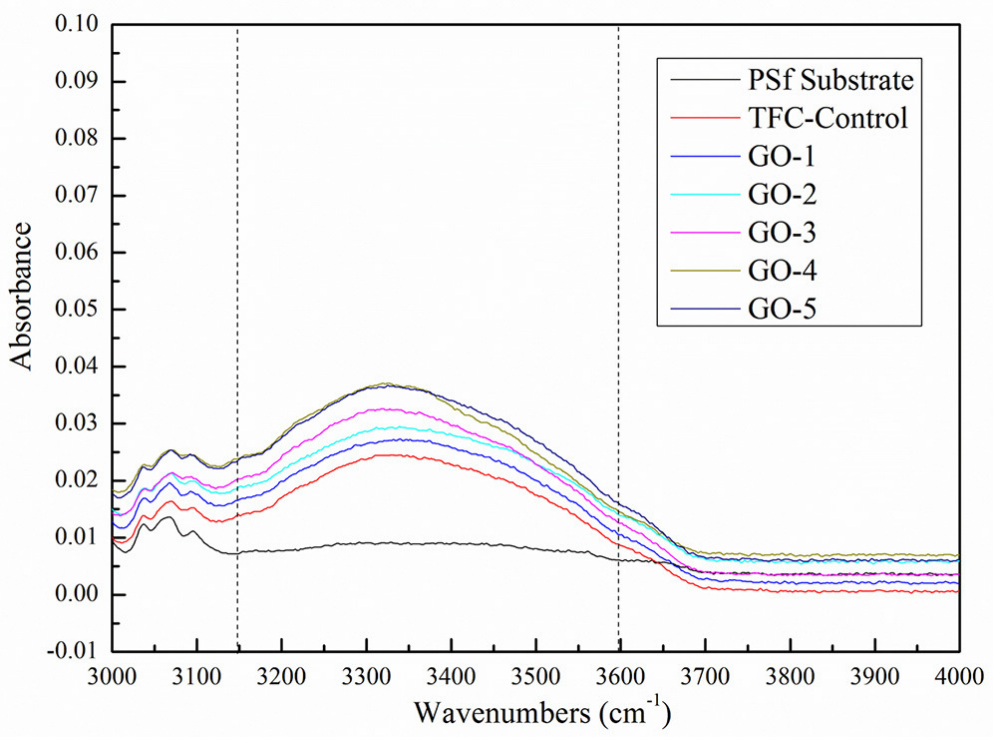
ATR-FTIR spectra of the as-prepared membranes.

The GO nano-sheets with hydroxyl functional groups in the aqueous phase are expected to improve the membrane surface hydrophilicity, as shown in Figure 5. The water contact angle of the PSf substrate was relatively high at 75.3°, but after the GO incorporation, the water contact angles of all the GO-incorporated membranes were improved, indicating the successful incorporation of GO nano-sheets in the PA layers. Moreover, the increase in the peak intensity of hydroxyl groups at about 3,100–3,600 cm^−1^ was also found in all the GO-incorporated TFC membranes, as shown in Figure 6, which are attributed to O–H groups stretching vibration in GO nano-sheets, leading to more hydrophilic PA membrane surfaces (Stobinski et al., 2014). However, for the GO-4 and GO-5 membranes with high GO nano-sheets concentrations, the water contact angles only decreased from 56.1 to 54.9°, which indicates too high concentrations of GO showed slight influence on the membrane surface hydrophilicity. The GO-incorporated membranes had higher hydrophilicity than the control sample might be related to the surface morphology and hydrophilic bulk of the membranes. Compared to the control membrane with a dense surface (see SEM image), the GO-incorporated membranes with a loose surface yielded a better wetting behavior likely due to the capillary effect. Apart from this, the GO-incorporated membranes had increased free volumes compared with the control membranes, which may be responsible for the improvement in the membrane wettability. Moreover, the GO-incorporated membranes with more O–H groups, leading to more hydrophilic PA membrane surfaces. As such, the GO-incorporated membranes had higher hydrophilicity than the control membrane.

### Membrane Desalination Performance

**Figure 7:**
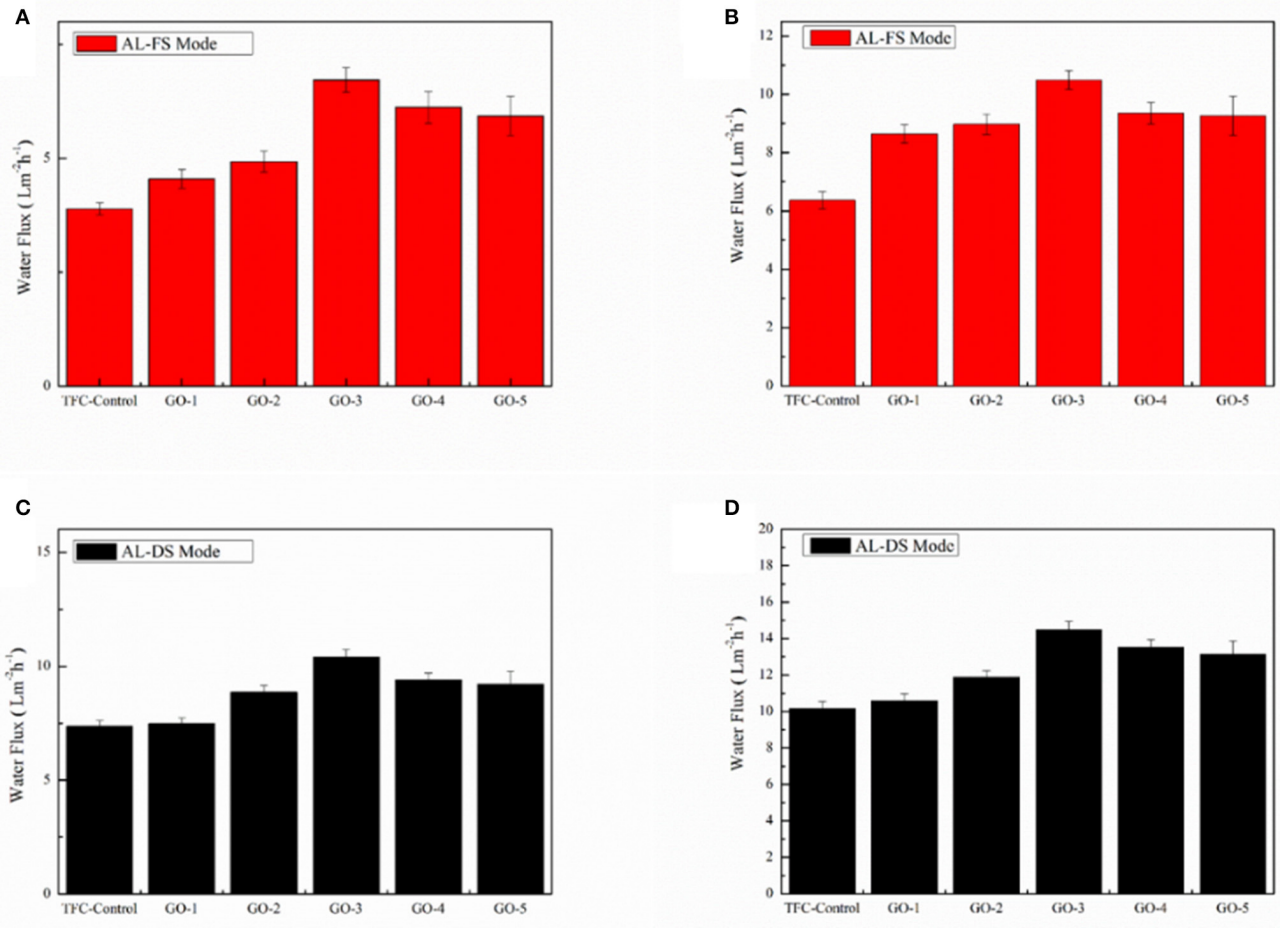
Water fluxes of the GO-incorporated TFC membranes in AL-FS mode: **(A)** 1.0 M NaCl of draw solution and **(B)** 2.0 M NaCl of draw solution, 2,000 ppm NaCl as feed solution. Water fluxes of the GO-incorporated TFC membranes in AL-DS mode: **(C)** 1.0 M NaCl of draw solution and **(D)** 2.0 M NaCl of draw solution, 2,000 ppm NaCl as feed solution.

Figure 7 shows the water fluxes of the FO membranes incorporated by different concentrations of GO nano-sheets in the AL-FS mode and the AL-DS mode with 1.0 and 2.0 M NaCl as draw solutions, respectively. It could be observed that the water fluxes of both AL-FS mode and AL-DS showed similar tendencies: with increasing concentrations of GO nano-sheets, the water fluxes initially went up and reached the optimal value typically being at 0.0100 wt%. Namely, the maximum water flux of GO-incorporated membrane was obtained when 0.0100 wt% of GO nano-sheets was added into the aqueous phase, with the water flux increasing 56.97% (from 7.32 to 11.49 L/m^2^h) in the AL-FS mode and 42.48% (from 10.17 to 14.49 L/m^2^h) in the AL-DS mode when using 2.0 M NaCl solution as the draw solution. This may be due to the improvement of the hydrophilic properties of GO-incorporated membranes. The higher the hydrophilic properties of the membranes, the easier for the water molecules to pass through the membranes (He et al., 2015; Xia et al., 2015; Shi et al., 2018). In addition, the decrease of the thickness of active PA layer of GO-incorporated membrane will also lead to an increase in FO water flux. However, when the GO nano-sheets concentration was too high, the membrane water flux began to decrease, which might be ascribed to the slight increase in the thickness of the active layer.

**Figure 8:**
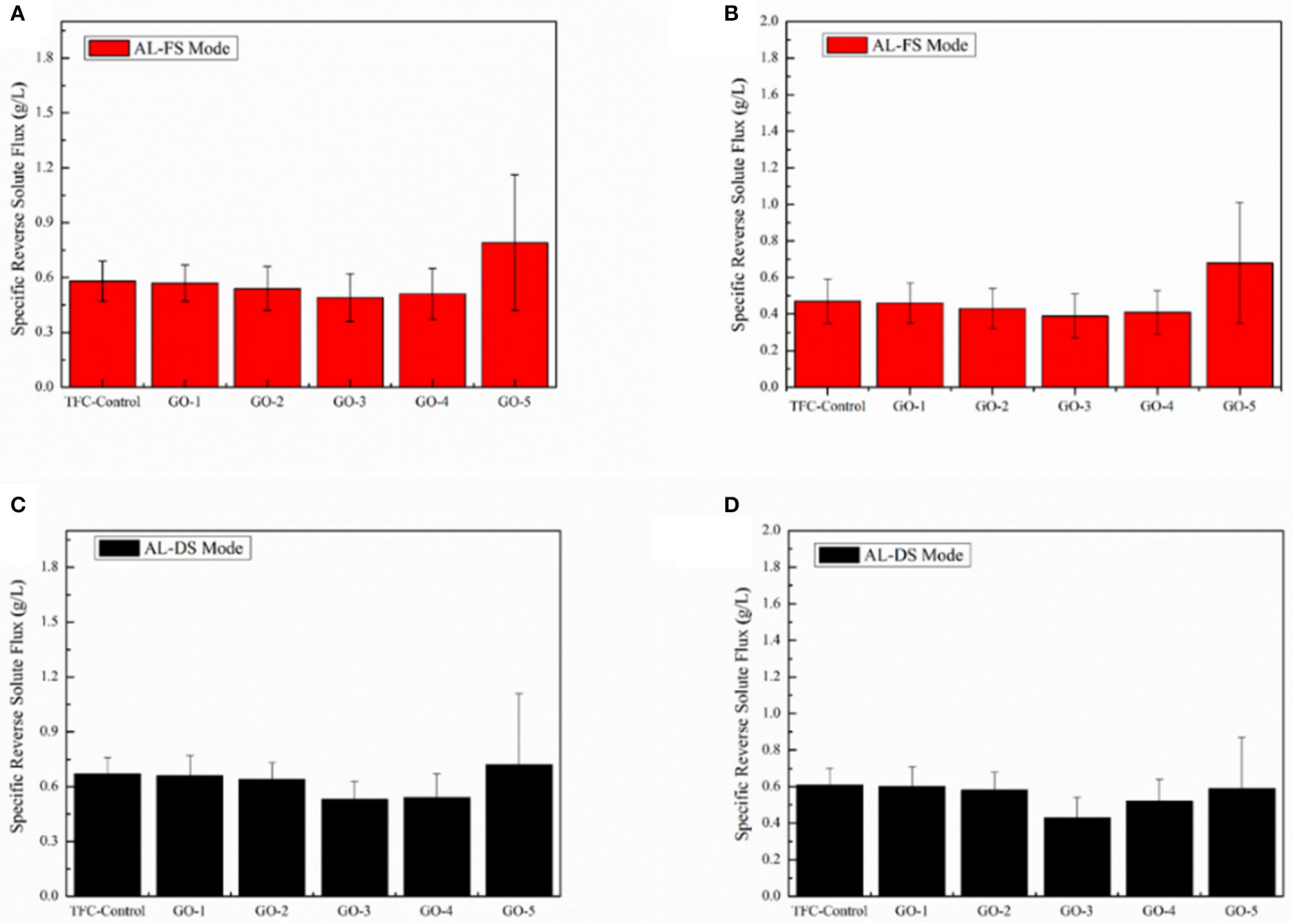
Specific reverse solute fluxes of the GO-incorporated TFC membranes in AL-FS mode: **(A)** 1.0 M NaCl of draw solution and **(B)** 2.0 M NaCl of draw solution, 2,000 ppm NaCl as feed solution. Specific reverse solute fluxes of the GO-incorporated TFC membranes in AL-DS mode: **(C)** 1.0 M NaCl of draw solution and **(D)** 2.0 M NaCl of draw solution, 2,000 ppm NaCl as feed solution.

Figure 8 shows the SRSFes of the FO membranes incorporated by different concentrations of GO nano-sheets in the AL-FS mode and the AL-DS mode with 1.0 and 2.0 M NaCl as draw solutions, respectively. The higher SRSF indicates relatively more solutes passing from the draw side to the feed side. Compared with the TFC-control membrane, the trend of SRSFes of the GO-incorporated membrane reduced and then increased along with increasing concentrations of GO nano-sheets. This is because the charge effect and two-dimensional capillary effect of GO nano-sheets, the water molecules pass easily in the PA layer in the meanwhile reject effectively salt ions. Therefore, moderate incorporation of GO nano-sheets can effectively improve the FO membrane selectivity. However, excessive loading of GO nano-sheets would lead to a defective surface of the membrane, resulted in the dramatically increase of GO-5 membrane SRSF. In addition, comparing the selectivity of the membrane with 1.0 and 2.0 M NaCl as draw solution, it can be seen that the selectivity of the membrane with 2.0 M NaCl was slightly higher than that of the former. This may be due to the increase of osmotic pressure resulting in a denser active layer.

### Membrane Separation and Transport Parameters

Table 2 studies the water permeability coefficient A, salt permeability coefficient B, and structural coefficient S value of GO-3 (the optimal membrane) and control membrane, respectively. The A value of control membrane was 0.206 LMHbar^−1^, while this value of GO-3 increased significantly to 0.278 LMHbar^−1^ when GO was introduced in aqueous phase. This trend agrees well with the results of FO desalination performance tests. The improvement of water permeability should be ascribed to the enhancement of GO-3 membrane hydrophilicity. Compared with the water permeability coefficient, the salt permeability coefficient of GO-3 decreased sharply to 0.009 LMH, which may be attributed to the interception of salt ions by GO nano-sheets. The trend of B value also agreed well with the behavior of SRSF. As for the membrane structure parameter, GO-3 membrane displayed 452 μm, only 57.7% of the control membrane, this may be caused by the PA layer thickness decrease (Zhang et al., 2013). In addition, A/B ratio is an important parameter to evaluate FO membrane selectivity, and higher A/B ratio indicates higher selectivity. The A/B ratio of GO-3 membrane showed 67.1% higher than that of the TFC-control membrane, which confirmed again the high desalination performance of the GO-3 membrane.

**Table 2:** Summary of the transport properties of the TFC–Control and GO-3 membrane.

**Names**	**A (LMHBar^**−1**^)**	**B-LMH**	**S (μm)**	**A/B (Bar^**−1**^)**	**R^**2**^ (J_**w**_)**	**R^**2**^ (J_**s**_)**	**CV (%)**
TFC-Control	0.206	0.116	784	1.776	0.973	0.965	1.58
GO-3	0.278	0.009	452	2.967	0.960	0.960	0.65

### Membrane Chlorine Resistance

**Figure 9:**
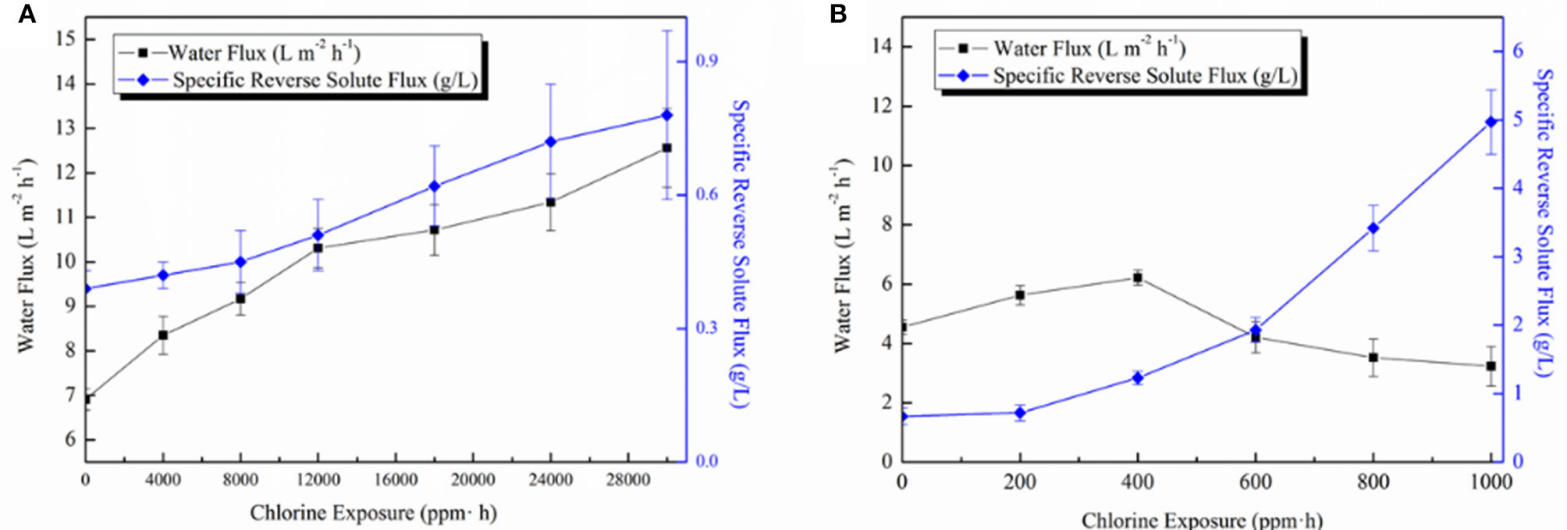
**(A)** The influence of chlorine immersion on the FO performance of GO-3 membrane and **(B)** The influence of chlorine immersion on the FO performance of the control membrane. The FO performance was tested in AL-FS mode with 1.0 M NaCl as the DS and DI water as the FS.

Membrane chlorine-resistance tests were conducted for both TFC-Control and GO-3 membranes as shown in Figure 9. For GO-3 membrane, a slow and mild increase of FO water flux as well as SRSF was observed during the chlorine-resistance test, which lasted up to as high as 30,000 ppm • h, before the SRSF value was doubled. The increased chlorine resistance of the GO-3 membrane might be attributed to hydrogen bonding between GO nano-sheets and PA layer blocking the replacement of amidic hydrogen with active chlorine. Moreover, the incorporated GO nano-sheets could keep the underlying PA chains from active chlorine attack. Therefore, a gradual increase of water flux due to the slow degradation of the PA layer was observed. At the same time, the surging rise of reverse solute flux was avoided because of the relatively compact and dense PA layer structure. As for the control membrane, the water flux increased initially but eventually dropped to 70.9% of the original flux in 1,000 ppm • h, while the SRSF greatly increased from 0.67 to 4.97 g/L. The amide bonds of the raw PA layers are vulnerable to free chlorine and can be easily destroyed by the N-chlorination, Orton rearrangement, and direct ring chlorination reactions, leading to the PA layer free volume increase as well the polymer matrix flexibility. Thus, at the beginning of the degradation of PA layer, the water molecules can easily transfer the PA layer, however, because of the fast degradation of the PA layer, then, a sharp increase of SRSF was observed, which caused a RO-like concentration polarization on top of the active layer thus decreasing both membrane water permeability and selectivity. Apparently, the GO-3 exhibited a much slower PA layer destruction when exposed to free chlorine, thus possessed good chlorine resistance characteristics (75.0 times enhancement).

## Conclusions

In this paper, a new type of TFC membranes was developed via using MPD/GO mixture as the aqueous phase for IP process. The GO nano-sheets were characterized by the FTIR, XRD, XPS, and SEM to confirm their successful syntheses. The GO-incorporated TFC membranes exhibited improved surface hydrophilicity and smoothness, as well as thin PA layer thickness, because of the affected diffusion rate of MPD monomers and increased aqueous phase solutions viscosity with the incorporation of GO nano-sheets. Compared with the control membrane, the desalination performance of the optimal membrane was significantly improved whereas only 0.0100 wt% GO nano-sheets was applied in this method. When 2.0 M NaCl was used as the draw solution, the water flux of GO-3 membrane increased 56.97% in AL-FS mode and 42.48% in AL-DS mode, at the same time, the SRSF of the membrane decreased indicated the selectivity of the membrane improved, which was due to the charge effect and two-dimensional capillary effect of GO nano-sheets made it easier for water molecules transfer and more effective for salt ions rejection. Therefore, the optimal GO-incorporated TFC membranes possessed higher water permeability, less salt permeability and lower structural parameter as compared to the control membrane. Moreover, the optimized membrane showed 75.0 times more chloride-resistant characteristics than the pristine membrane due to hydrogen bonding between GO nano-sheets and PA layer and the GO nano-sheets protection. The excellent water permeability and chloride resistance of this new type of membrane could facilitate wider applications of FO membranes in desalination applications.

## Data Availability Statement

All datasets generated for this study are included in the article.

## Author Contributions

All authors extensively discussed the results, reviewed the manuscript, and approved the final version of the manuscript to be published.

### Conflict of Interest

The authors declare that the research was conducted in the absence of any commercial or financial relationships that could be construed as a potential conflict of interest.
